# Baicalein suppresses the androgen receptor (AR)-mediated prostate cancer progression *via* inhibiting the AR N-C dimerization and AR-coactivators interaction

**DOI:** 10.18632/oncotarget.22319

**Published:** 2017-11-06

**Authors:** Defeng Xu, Qiulu Chen, Yalin Liu, Xingqiao Wen

**Affiliations:** ^1^ School of Pharmaceutical Engineering and Life Sciences, Changzhou University, Changzhou, Jiangsu 213164, P.R. China; ^2^ Department of Urology, Shenzhen Hospital of Southern Medical University, Shenzhen, Guangdong 518100, P.R. China

**Keywords:** anti-androgen, baicalein, flavonoids, prostate cancer, androgen receptor

## Abstract

**Background:**

Androgen receptor (AR) plays a critical role in prostate cancer (PCa) development and progression. Androgen deprivation therapy with antiandrogens to reduce androgen biosynthesis or prevent androgens from binding to AR are widely used to suppress AR-mediated PCa growth. However, most of ADT may eventually fail with development of the castration resistance after 12-24 months. Here we found that a natural product baicalein can effectively suppress the PCa progression *via* targeting the androgen-induced AR transactivation with little effect to AR protein expression.

**Methods:**

PCa cells including LNCaP, CWR22Rv1, C4-2, PC-3, and DU145, were treated with baicalein and luciferase assay was used to evaluate their effect on the AR transactivation. Cell growth and IC_50_ were determined by MTT assay after 48 hrs treatment. RT-PCR was used to evaluate the mRNA levels of AR target genes including PSA, TMPRSS2, and TMEPA1. Western blot was used to determine AR and PSA protein expression.

**Results:**

The natural product of baicalein can selectively inhibit AR transactivation with little effect on the other nuclear receptors, including ERα, and GR. At a low concentration, 2.5 μM of baicalein effectively suppresses the growth of AR-positive PCa cells, and has little effect on AR-negative PCa cells. Mechanism dissection suggest that baicalein can suppress AR target genes (PSA, TMPRSS2, and TMEPA1) expression in both androgen responsive LNCaP cells and castration resistant CWR22Rv1 cells, that may involve the inhibiting the AR N/C dimerization and AR-coactivators interaction.

**Conclusions:**

Baicalein may be developed as an effective anti-AR therapy via its ability to inhibit AR transactivation and AR-mediated PCa cell growth.

## INTRODUCTION

Prostate cancer (PCa) is the second leading cause of cancer death among American men. In 2015, approximately 228,800 men were diagnosed with PCa, and 27,540 men were expected to die from this disease in the United States [1]. The lifetime risk for a U.S. male to develop PCa is about 1 in 6, although the risk of dying from PCa is only 1 in 35 [2]. Use of androgen deprivation therapy (ADT) as a therapeutic modality for PCa patients didn't start until 1941 when Huggins and Hodges treated advanced PCa patients via ADT with surgical castration [3]. Since then, ADT has been proven to be as an effective treatment for patients with advanced PCa.

ADT aims to reduce the biosynthesis of androgens and/or prevent from androgen binding to androgen receptor (AR) [4–6]. However, most patients relapse after an initial response to ADT, and eventually develop the castration-resistant prostate cancer (CRPC) [7, 8]. Mechanism dissection indicated that the failure of ADT might involve the inability to further suppress the AR-mediated PCa progression, and many of ADT might lead to increase the expression of AR even the androgen concentration was maintained at the castration level [9, 10]. Importantly, recent studies also suggested that non-androgens including various growth factors, cytokines, protein kinases or interacting with selective coactivators could also transactivate AR in an androgen-independent mechanism [11]. Targeting AR, instead of targeting the androgens with antiandrogens, to better suppress the AR-mediated CRPC progression is urgently needed [12, 13].

Flavonoids are widely used in the Chinese herbal medicines, and epidemiological studies indicated that an increased intake of dietary flavonoids might be associated with a decreased risk of selective cancers [14–16]. Baicalein (5,6,7-trihydroxyflavone) is a bioactive flavonoid originally isolated from the root of *Scutellaria baicalensis Georgi* [17], that has been widely used to treat with various inflammatory diseases including cardiovascular diseases, chronic hepatitis [18–21] and some selective cancers including breast cancer, hepatocellular carcinoma, leukemia, and colon cancer [22–26].

Here we investigated the ability of baicalein to regulate AR transactivation, and results revealed that baicalein could inhibit the growth of PCa AR-positive cells including LNCaP, C4-2 and CWR22Rv1 cells, with little effect on the AR-negative PC-3 and DU145 cells. Mechanism dissection indicated that baicalein could effectively inhibit AR activity via inhibiting the AR dimerization and AR-coregulation complex formation.

## RESULTS

### Baicalein specifically inhibits the DHT-mediated AR transactivation, but not the ER, PR, and GR-mediated transactivation

**Figure 1:**
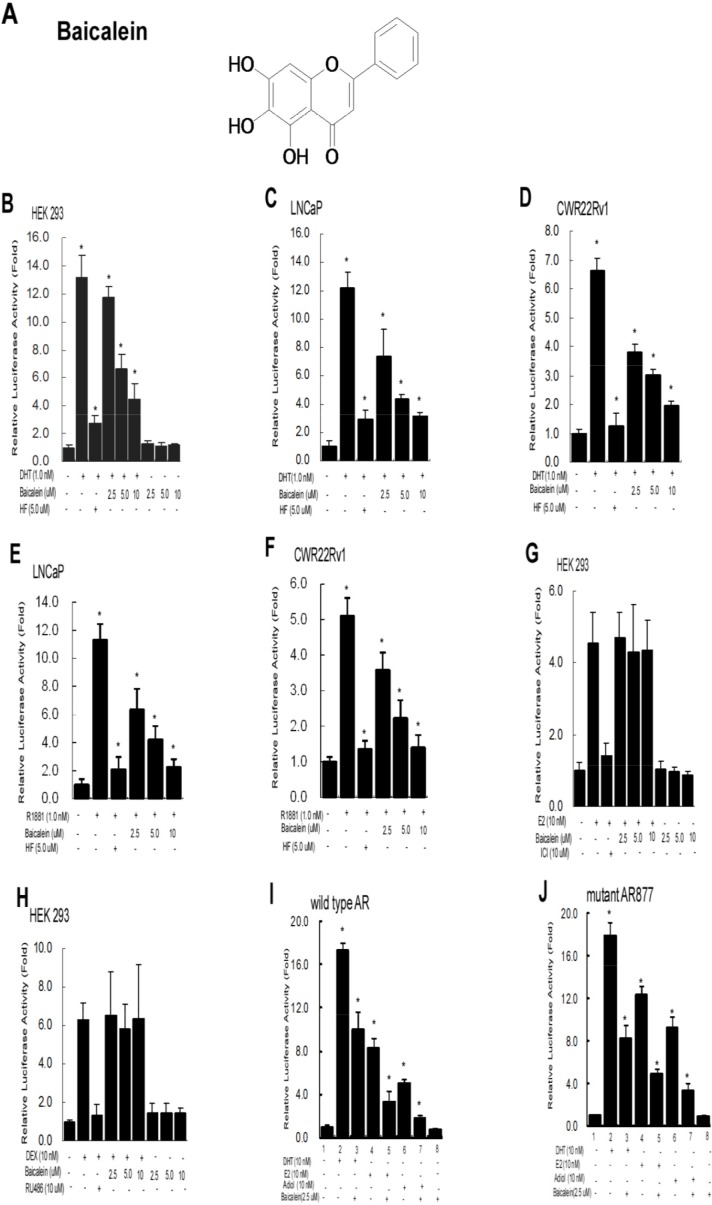
Baicalein selectively inhibits DHT-mediated AR transactivation, but not the ERα, PR, or GR activity **(A)** Chemical structure of Baicalein. **(B)** Baicalein inhibits the androgen-induced transcriptional activity of wild type AR in HEK 293 cells. **(C)** Baicalein inhibits the DHT-induced AR transcriptional activity of a gain-of-function mutant AR (T877A) in prostate cancer LNCaP cells. **(D)** Baicalein inhibits the DHT-induced AR transcriptional activity in CWR22Rv1 cells. **(E)** Baicalein inhibits the R1881-induced AR transcriptional activity of a gain-of-function mutant AR (T877A) in prostate cancer LNCaP cells. **(F)** Baicalein inhibits the R1881-induced AR transcriptional activity in CWR22Rv1 cells. **(G-H)** Baicalein shows no effect on the transcriptional activities of estrogen-induced ERα and DEX-induced GR in HEK 293 cells. MMTV-Luc or ERE-Luc activities were determined. **(I-J)** Baicalein inhibits the E2 and Adiol-induced full length AR transactivation. AR-regulated MMTV-Luc reporter gene was activated in the presence of 10 nM DHT, E2, or Adiol in HEK293 cells (lanes 2, 4, 6). 5 μM Baicalein could effectively inhibit the DHT, E2, and Adiol-stimulated AR activity (lanes 3, 5, 7). The solvent (DMSO) treated AR-baseline transcriptional activity was counted as 1 fold (lane 1). Data were averaged from three independent experiments.

Early studies indicated Baicalein (see its structure in Figure 1A) might suppress several inflammatory diseases and some selective cancers [18–26]. Its potential effect to the PCa progression *via* impacts on the AR dimerization and AR transactivation, however, remains unclear. We first examined its effect on the AR transactivation *via* assaying the luciferase activity with MMTV containing the androgen-response-element (ARE) in the HEK 293 cells, and results revealed that androgen-DHT induced AR transactivation was suppressed but by antiandrogen-HF (Figure 1B). Interestingly, adding baicalein also led to suppress the DHT-induced AR transactivation (Figure 1B). Similar results were also obtained when we replaced HEK 293 cells with androgen-sensitive PCa LNCaP cells and CRPC CWR22Rv1 cells, showing 1 nM DHT-induced AR transactivation was suppressed by baicalein in a dose-dependent manner (Figure 1C-1D). Consistently, the AR activity induced by an alternative ligand, R1881, can also be inhibited by baicalein treatment (Figure 1E, 1F). In contrast, we found baicalein failed to suppress the 10 nM estrogen (E2)-induced ERα transactivation (Figure 1G) and 10 nM glucocorticoid-Dex-induced GR transactivation (Figure 1H).

We further studied the effects of baicalein on the other inducers including D5-androstenediol (Adiol) or E2 to the AR transactivation [36], and results revealed that 5 μM baicalein could effectively inhibit the Adiol- or E2-mediated transactivation of wild type (Wt) AR and mutant AR in HEK 293 cells (Figure 1I-1J).

### Baicalein treatment inhibits the DHT-induced growth of AR-positive, but not the AR-negative PCa cells

**Figure 2:**
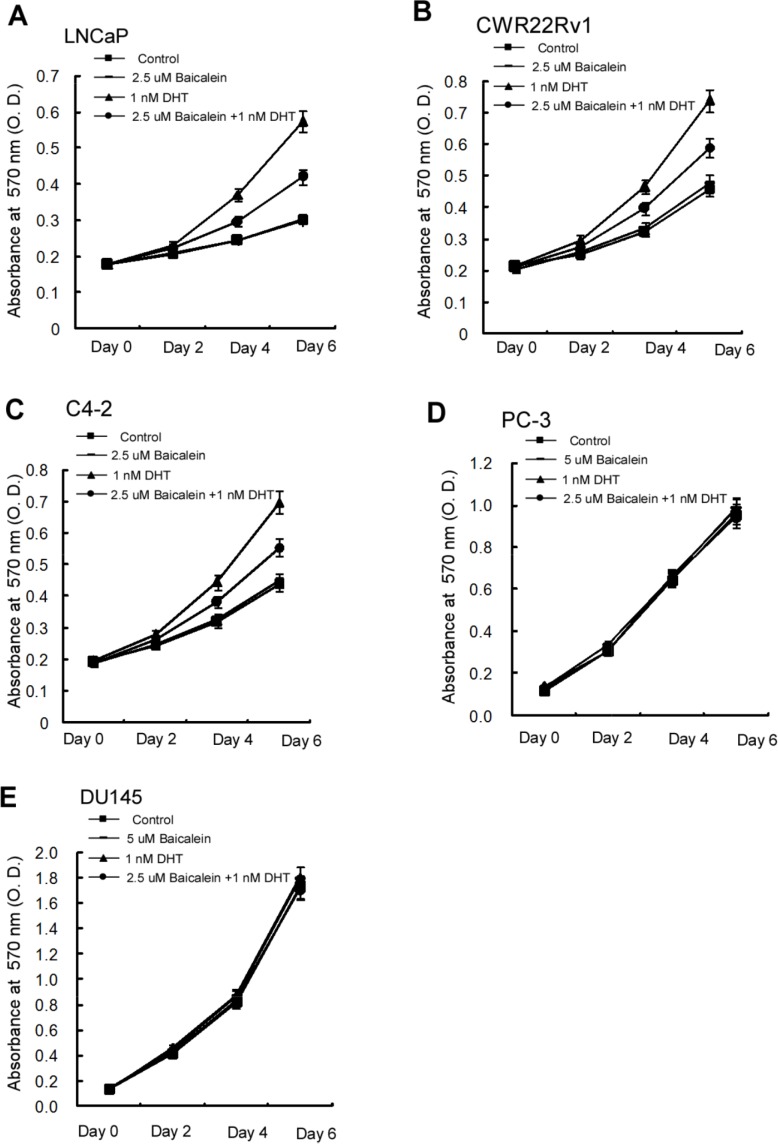
Differential growth inhibition effects of Baicalein on different prostate cells Cells were treated with dimethyl sulphoxide and baicalein (5 μM) in the absence or presence of 1.0 nM DHT. Media with indicated treatments were refreshed every 2 days for a total of 7 days. **(A)** Baicalein inhibits the DHT-induced growth of LNCaP cells **(B)** Baicalein inhibits the DHT-induced growth of CWR22Rv1 cells. **(C)** Baicalein inhibits the DHT-induced growth of C4-2 cells. **(D)** No significant effect of baicalein on the growth of AR-negative PC-3 cells. **(E)** There was no effect of baicalein on the growth of AR-negative DU145 cells. Data represent mean ± SD of three independent experiments with three replicates in each experiment.

To further study the consequences of baicalein-suppressed AR transactivation, we investigated its impact on the AR-mediated PCa cell growth. The results from MTT growth assay suggested that the 2.5 μM baicalein could effectively reduce DHT-induced cell growth in androgen-sensitive LNCaP cells by 56.5%, as well as CRPC CWR22Rv1 cells by 48.5% and C4-2 cells by 51.5% (Figure 2A-2C). In contrast, baicalein has little effect on the growth of PCa AR-negative cells including PC-3 and DU145 cells (Figure 2D-2E).

**Figure 3:**
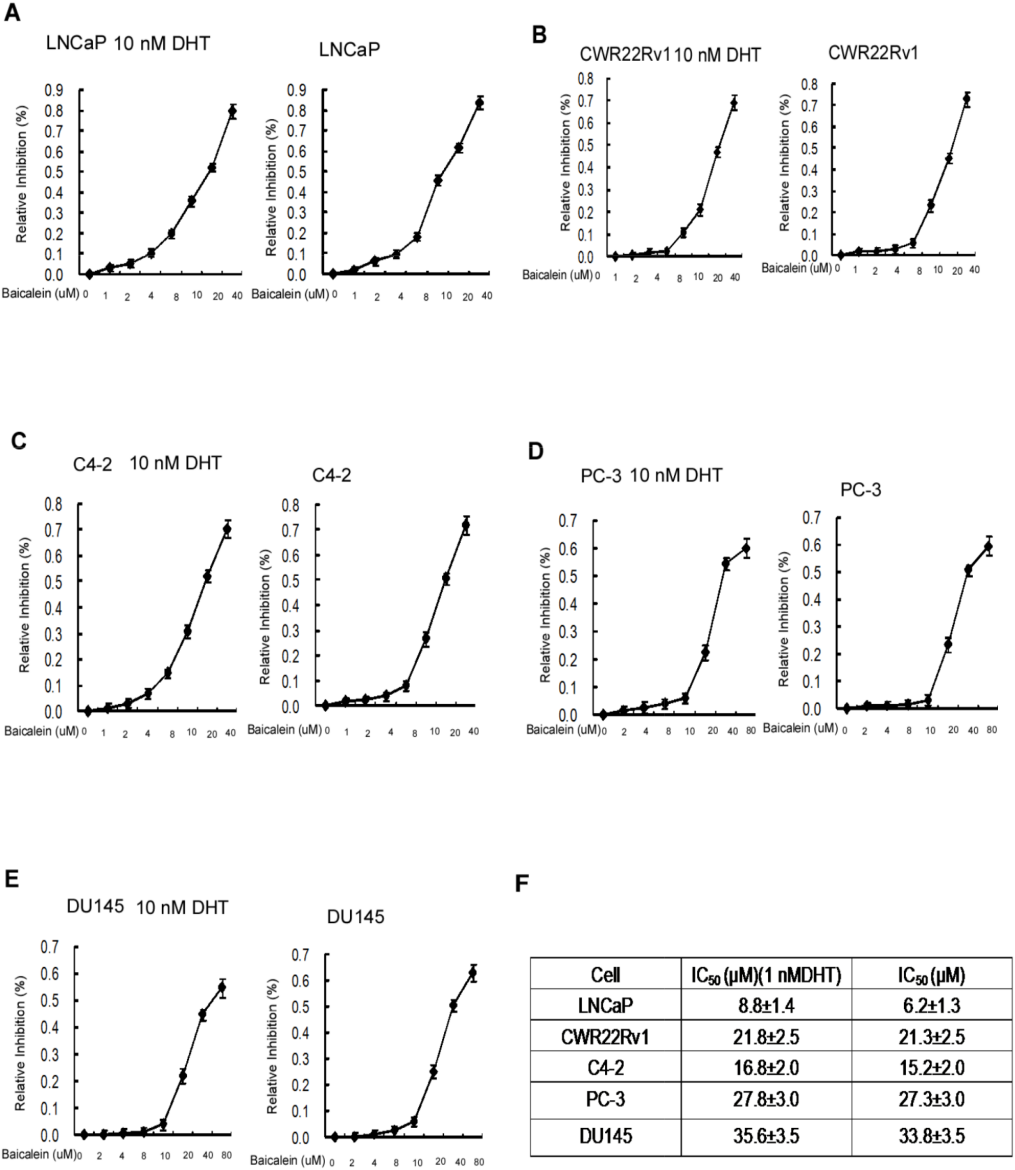
IC50 of Baicalein in different prostate cancer cells We determined the cell half-inhibition (IC50) of Baicalein in LNCAP, CWR22rv1, C4-2, PC-3, and DU145 cells. Cells were seeded on 24-well plates in media with 10%FBS for 24 hrs. Media were then refreshed to media with 10% CS-FBS for another 24 hrs, and cells were treated with serial concentrations of baicalein with or without 10 nM DHT for 48 hrs. Cell growth and IC_50_ value were determined by MTT assay. Half-inhibition of baicalein is shown in Figure **3A**–**3E.** Data represent mean ± SD of two independent experiments with three replicates in each experiment.

The half maximal inhibitory concentration (IC50), a measure of the effectiveness of a compound in inhibiting biological or biochemical functions [37], for baicalein on the PCa cells in the presence of DHT in LNCaP, CWR22Rv1, C4-2, PC-3, and DU145 were 8.8±1.4 μM, 21.8±2.5 μM, 16.8±2.0 μM, 27.8±3.0 μM and 35.0±3.5 μM, respectively (Figure 3A-3E). In the absence of DHT, the IC_50_ concentrations for baicalein in LNCaP, CWR22Rv1, PC-3 and DU145 were 6.2±1.3 μM, 21.3±2.5 μM, 15.2±2.0 μM, 27.3±3.0 μM and 33.8±3.5 μM, respectively (Figure 3A-3E). Although it seems the IC50 values (Figure 3) for the various PCa cells do not correlate with the levels of growth inhibition (Figure 2), there is no conflict between the presented Figure 2 and Figure 3 data. One difference is the experimental condition: the IC50 of Figure 3 was determined after 48 hours (2 days) of compound treatment and the Figure 2 baicalein growth inhibition data were determined by continual treatment of 2, 4, and 6 days. The determination of IC50 values after 2 days treatment is a generally agreed upon protocol.

**Figure 4:**
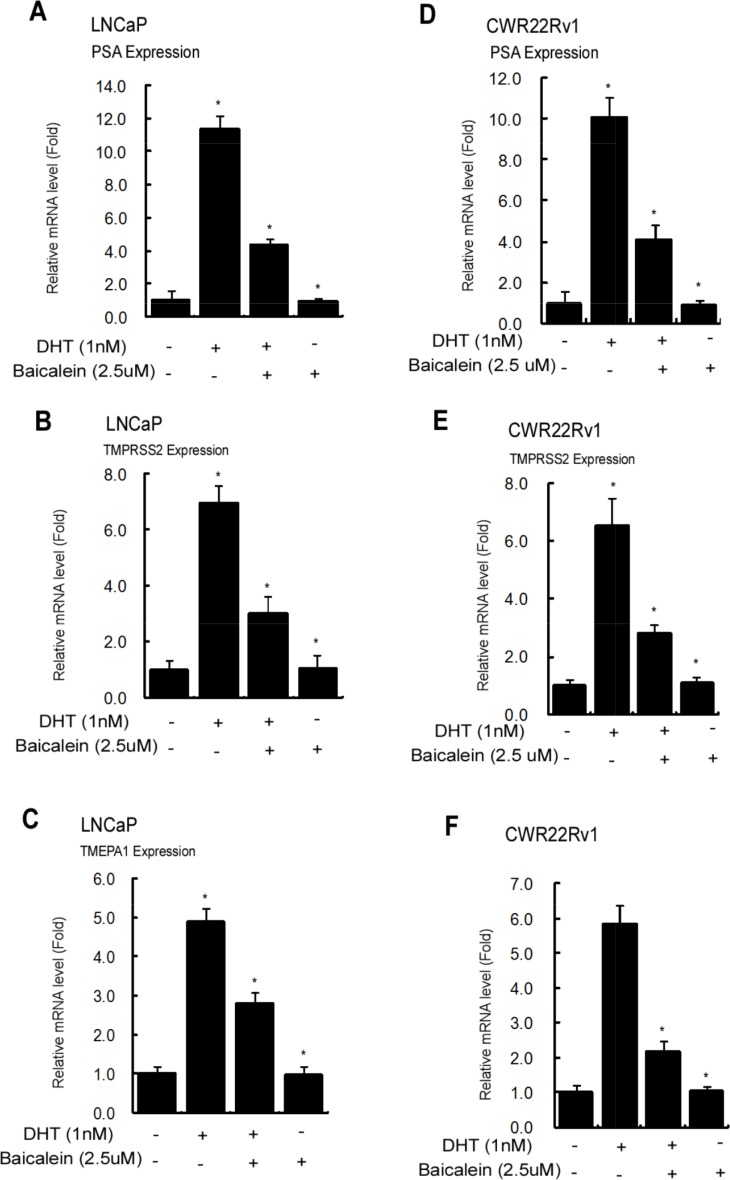
Baicalein Inhibits the AR target gene expression in LNCaP and CWR22Rv1 cells Cells were treated with dimethyl sulphoxide or baicalein (5 μM) in the absence or presence of 1 nM DHT for 24 hrs. We used real-time Q-PCR to analyze the mRNA expressions of AR target genes, PSA, TMPRSS2, and TMEPA1, in LNCaP and CWR22Rv1 cells. The respective mRNA level of these genes in each treatment group was displayed as fold changes compared to the untreated group. Data are shown as the mean ± SD of three independent experiments with triplicates in each experiment.

Together, results from Figure 2–3 suggest that baicalein may suppress the PCa AR-positive cell growth. This conclusion is further supported by the results from qPCR assay showing baicalein can suppress the 1 nM DHT-induced expressions of several AR target genes including PSA, TMPRSS2, and TMEPA1 in a dose-dependent manner in LNCaP and CWR22Rv1 cells (Figure 4).

### Mechanism dissection how Baicalein can suppress AR transactivation

**Figure 5:**
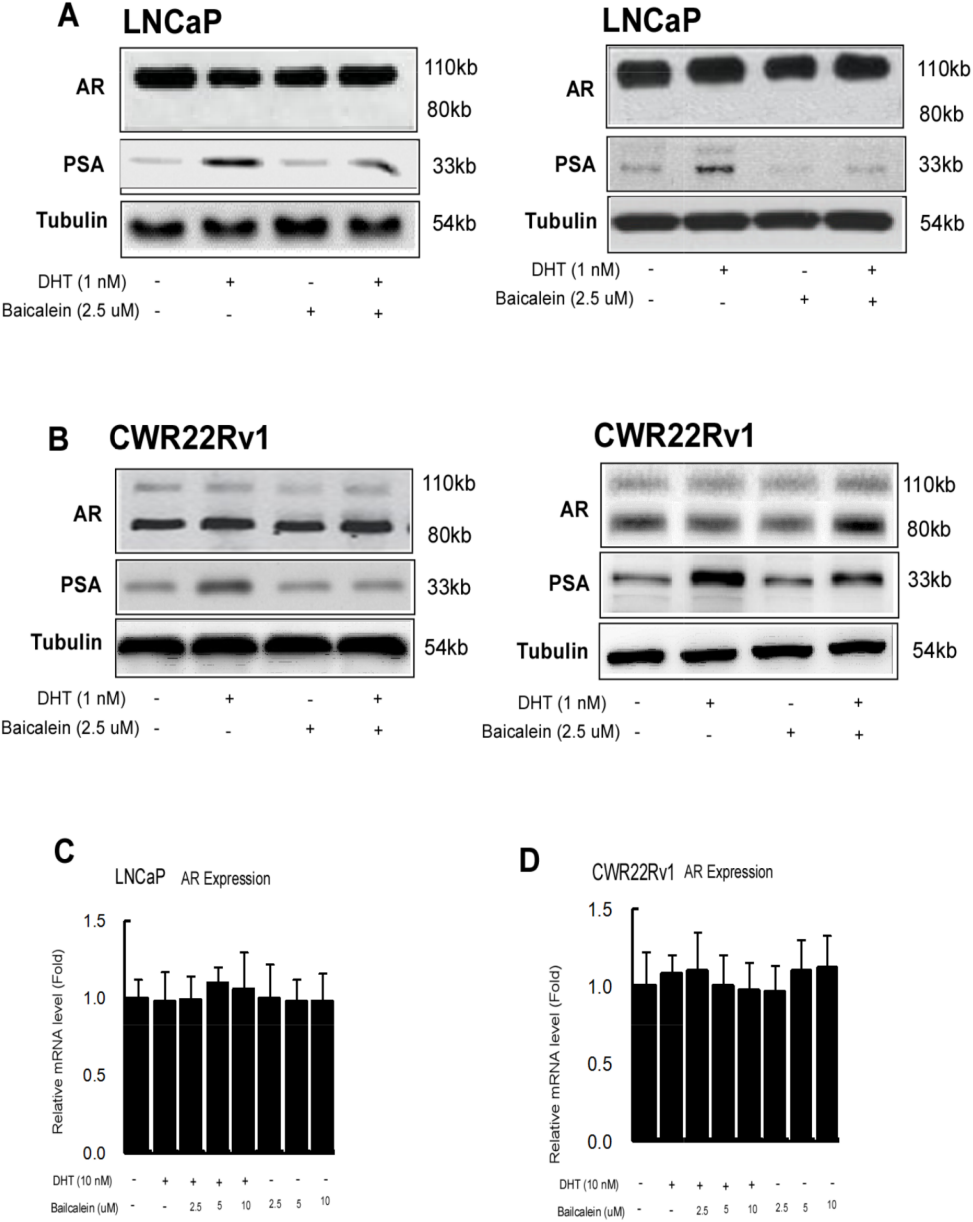
Baicalein inhibited AR transactivation is not via changing AR protein expression or stability **(A** and **B)** Western blot analyses of PSA and AR levels in control and baicalein treated LNCaP or CWR22Rv1 cells in the absence or presence of DHT. Fifty μg of total protein from cells was applied onto a 10% sodium dodecylsulfate-polyacrylamide gel and subjected to electrophoresis followed by Western blot using anti-AR, anti-PSA antibodies, and anti-tubulin antibodies. The values below the figures represent change in density of the bands normalized to α-tubulin. Representative graphs from two independent experiments were shown. **(C** and **D)** Baicalein does not affect AR gene expression mRNA levels in LNCaP and CWR22Rv1 cells. Cells were treated with dimethyl sulphoxide or various concentrations of Baicalein in the absence or presence of 1 nM DHT for 24 hrs. We used real-time Q-PCR to analyze the AR mRNA expressions in LNCaP and CWR22Rv1 cells. Data are shown as the mean ± SD of three independent experiments with triplicates in each experiment.

To dissect the mechanism how baicalein can suppress AR transactivation, we first examined its effect on AR expression, and results from Western blot analysis revealed that 2.5 μM baicalein had little effect on the AR protein expression in the LNCaP and CWR22Rv1 cells in the presence of 1 nM or 10 nM DHT (Figure 5A-5B). In contrast, 2.5 μM baicalein suppressed the DHT-induced PSA protein expressions (Figure 5A-5B).

**Figure 6:**
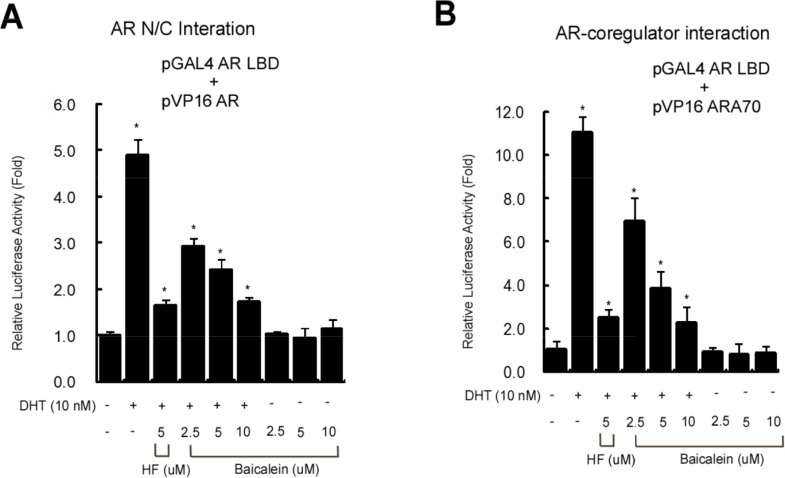
**(A)** Baicalein inhibits the AR N-C dimerization using mammalian 2-hybrid interaction assay. **(B)** Baicalein inhibits the interaction of AR and AR coregulator using a mammalian 2-hybrid assay. Data are shown as the mean ± SD of three independent experiments with triplicates in each experiment.

We then studied its effect on the AR N-terminal and C-terminal (N-C) interaction that may play key role for AR functions [38, 39], and results from the mammalian 2-hybrid interaction assay in cells transfected with GAL4-RE-Luc, GAL4-DBD fused-AR LBD (GAL4-AR LBD), and VP16 fused AR (VP16-AR) revealed that DHT could stimulate the AR N-C interaction, and baicalein could suppress AR N-C interaction in a dose-dependent manner (Figure 6A).

In addition to the AR dimerization, results from mammalian 2-hybrid assay also revealed that baicalein, like HF, could suppress the DHT-induced interaction of AR and its coactivator ARA70 [40] (Figure 6B).

Together, results from Figure 6A-6B suggest baicalein may suppress the AR transactivation *via* inhibiting the AR N/C dimerization and AR-coactivator interaction.

## DISCUSSION

Traditional Chinese Medicine has been used increasingly as complementary medicine in cancer care [41]. Studying potential value of herbal medicines in clinical care has been encouraged by the World Health Organization's Traditional Medicines Strategy [42].

Flavonoids are a subclass of polyphenolic compounds that have been widely used for thousands of years in Oriental medicine due to their anti-inflammatory, anti-oxidants and anti-microbial effects [43–45]. Recently, flavonoids have been demonstrated to exhibit antitumor effects in various types of cancer [46–48].

Despite the effectiveness of ADT and anti-androgen treatment strategies to delay the progression of PCa, many patients still develop the CRPC and thus there is an urgent need for alternative therapeutic targets to AR [49]. Most CRPC continues to be dependent on transcriptionally active AR for cell proliferation and survival, and it can be activated by several non-androgens factors in the castration androgen environment, suggesting in addition to use antiandrogens to reduce or prevent androgens binding to AR, targeting AR with other factors might present another approach to further suppress these CRPC [50]. Using baicalein to suppress DHT-induced AR transactivation *via* altering the AR N/C dimerization and AR-coactivator interaction is one of such approaches.

Recent studies indicated ADT with newly developed antiandrogen Enzalutamide might induce the castration resistance *via* inducing the AR splicing variant ARv7 [51–53]. Since all current antiandrogens failed to suppress ARv7 transactivation due to lack of the androgen-binding-domain, it will be interesting to see if baicalein can also suppress the interaction between ARv7 and AR.

## MATERIALS AND METHODS

### Reagents

Commercial compounds and reagents include 5α-dihydrotestosterone (DHT), dexamethasone (DEX), RU486, progesterone, hydroxflutamide (HF), 17β-estradiol (E2), ICI 182,780 (ICI), methanol, dimethyl sulphoxide (DMSO), chloroform (CHCl_3_), and [3-(4,5- Dimethylthiazol-2-yl)-5-(3-carboxyme-thoxyphenyl)-2-(4-ulfophenyl)-2H-tetrazolium] (MTT) were purchased from Sigma-Aldrich (St Louis, MO, USA) and stored at −20°C in the dark. All other chemicals and solvents used in this study were of reagent grade or high performance liquid chromatography (HPLC) grade.

### Plant extracts preparation

Fresh whole plants of *Scutellaria baicalensis Georgi* were purchased from a Chinese medicinal herb market in Jiangsu. Whole air-dried roots of *Scutellaria baicalensis Georgi* (500 g) were extracted with 70% ethanol at effluent temperature for three hrs twice. The solvent was evaporated to obtain crude extract (49.8g), which was applied to the silica gel column chromatography, eluted by n-Hexane- Ethyl acetate mixture and petroleum ether- Ethyl acetate as mobile phases to obtain different fractions, based on the TLC pattern. Baicalein (28 mg) was separated and purified under silica gel column chromatography with a solvent system of petroleum- Ethyl acetate mixture, and identified by comparison of NMR and MS spectral data with reference values [27, 28]. The stock solution of baicalein for incubation with cells was prepared in dimethyl sulfoxide and further diluted in culture medium. Final concentration of DMSO in the medium was 0.1% (to avoid its interference with cell viability). H^1^NMR: (δ, ppm, DMSO-d_6_, 400MHz):12.57 (2, 1H,), 7.82-7.79 (m, 2H), 7.49 (d,1H, J=15.2Hz), 6.64 (d, 1H, J=15.2Hz), 3.08(m, 2H), 2.26 (s, 3H), 1.72 (m,2H), 1.62 (m, 2H), 1.27 (s, 6H).

### Cell culture

Cells were cultured at 5% CO_2_ and 37°C. LNCaP is an androgen-responsive and androgen dependent-human PCa cell line with a mutant AR (T877A); CWR22Rv1 is an androgen-responsive but androgen-independent human PCa cell line, which expresses endogenous AR; C4-2 cell line, is androgen-independent, has increased sensitivity to low levels of androgens; DU145 and PC-3 are androgen-independent human PCa cell lines that lack expression of AR. LNCaP, CWR22Rv1, C4-2, PC-3, and DU145 cell lines were maintained in RPMI-1640 medium containing 10% fetal bovine serum (FBS) and antibiotics (100 units/mL penicillin, 100 μg/mL streptomycin).

HEK 293 cell line was generated by transformation of human embryonic kidney cell cultures, and maintained in Dulbecco's modified Eagle's medium (Life Technologies) supplemented with 10% FBS and the above antibiotics.

### Plasmids

The plasmids used were pSG5AR, full-length cDNA of wild-type human AR, MMTV-Luc (MMTV) a luciferase reporter plasmid, pSG5 progesterone receptor (pSG5PR), pSG5 glucocorticoid receptor (pSG5 GR), PIRES-flag-ARt877a, pSG5, pSG5AR(N-DBD), pcDNA3.1-ERα, pGL3 (ERE)_3_-Luc, pRL-TK pCMX-VP16-ARA70, Gal4-AR-LBD, and Gal4-RE-Luc, all of which were constructed as described [29–32].

### Luciferase assays

Luciferase activity, transfections, and reporter gene assays, were performed by using Lipofectamin 2000 (Invitrogen) according to the manufacturer's protocol. HEK 293 cells, lacking functional AR or ERα, were transfected with wild-type AR or ERα expression plasmid and reporter gene. Briefly, 2×10^4^ HEK 293 cells were plated on 24-well dishes with 10% charcoal stripped (CS)-FBS DMEM medium for 24 hrs, medium was refreshed, and cells were transfected with pSG5-AR, pSG5-PR, pSG5-GR (for AR, PR, or GR transfections, respectively), MMTV-Luc, and pRL-TK-Luc, or with cDNA3.1-ERα, pGL3 (ERE)_3_-Luc, and pRL-TK-Luc for ERα transfections for 24 hrs. After transfection, the medium was refreshed to 10% CS-FBS medium and cells treated with various concentrations of baicalein in the presence or absence of 1 nM DHT and/or 5 μM HF for 24 hrs for AR transfections, and treated with a serial concentrations of baicalein or 10μM anti-estrogen (ICI 182,780) in the absence or presence of 10 nM E2 for 24 hrs for ER transfections. For the PR and GR reporter activity assay, 10 nM progesterone, or DEX were added, respectively. To inhibit the progesterone-PR or DEX-GR activities, 10 μM RU486 was added.

Briefly, 4×10^4^ LNCaP cells or CWR22Rv1 cells were plated on 24-well dishes with 10% CS-FBS RPMI-1640 medium for 24 hrs, medium was refreshed and cells transfected with MMTV-ARE-Luc and pRL-TK-Luc for 24 hrs. After transfection, the medium was changed to 10% CS-FBS medium for treatment with various concentrations of baicalein in the presence or absence of 1 nM DHT and/or 5 μM HF for 24 hrs. These cells were then harvested and assayed for luciferase activity using the Dual Luciferase Assay System. Data were expressed as relative luciferase activity normalized to the internal Renilla luciferase control.

For the mammalian 2-hybrid assay to determine the AR N-C interaction and AR-AR coregulator interaction, HEK 293 cells were plated on 24-well dishes with 10% CS-FBS DMEM medium for 24 hrs. Cells were transfected with pGal4-RE-Luc reporter plasmid, pGal4-ARDBD-LBD (AR DNA binding domain and ligand binding domain), pCMX-VP16-AR or pCMX-VP16-ARA70 plasmids as indicated in the figure. After 24 hrs transfection, the medium was refreshed to 10% CS-FBS medium and cells were treated with 1 nM DHT and/or baicalein for an additional 24 hrs. tk-RL luciferase was co-transfected as the internal control. Cells were then harvested for the dual luciferase assay (Promega, WI).

### RT-PCR and real-time PCR

Total RNA was extracted from PCa cells using Trizol (Invitrogen). Reverse transcription was performed using the Superscript first-stand synthesis kit (Invitrogen). Quantitative real-time PCR analyses using the comparative CT method were performed on an ABI PRISM 7700 Sequence Detector System using the SYBR Green PCR Master Mix kit (Perkin Elmer, Applied Biosystems, Wellesley, MA, USA) according to the manufacturer's instructions. After an initial incubation at 50°C for 2 min and 10 min at 95°C, amplification was performed for 40 cycles at 95°C for 20 s, 65°C for 20 s, and 72°C for 30 s. Specific primer pairs were determined with the Primer-Express program (Applied Biosystems). The PSA primer pairs were 5′-AGG CCT TCC CTG TAC ACC AA-3′ and 5′-GTC TTG GCC TGG TCA TTT CC-3′. The TMPRSS2 primer pairs were 5′-GTA CAC TGT TTC CAT GTT ATG-3′ and 5′-AAT AAG AAG GAG TCA TTT GAG-3′. The TMEPA1 primer pairs were 5′-CCT TCT CTT CCC CTT TCC ATC TCC-3′ and 5′-GTC CCG CCA ACC CCA AAT CTA TCT-3′. The hAR primer pairs were 5′-AAG GAT GGA AGT GCA GTT AG-3′ and 5′-GT CCA CCG GGT TCT CCA GCT-3′. The normalization control used was β-actin, and the primers used were 5′-TCA CCC ACA CTG TGC CCC ATC TAC GA-3′ and 5′-CAG CGG AAC CGC TCA TTG CCA ATG G-3′.

### Western blot analysis

2.5×10^6^ cells (per 100-mm dish) were washed with 1x PBS and scraped into a lysis buffer. Protein concentrations were measured with the BCA protein reagent (Pierce Chemical, Rockford, IL). Approximately 50 μg of protein/lane were loaded and run on a 10% polyacrylamide gel with a Tris/glycine running buffer system and then transferred onto a polyvinylidene difluoride membrane. The blots were probed with primary anti-AR (N-20), anti-PSA (C-19) antibodies with dilutions of 1:500 to 1:1,000 and incubated at room temperature for 2 hrs. The secondary antibody [rabbit anti-goat IgG, 1:5,000 dilution (Santa Cruz Biotechnology) or rabbit anti-mouse IgG, 1:5,000 dilution (Pierce Chemical, Rockford, IL)] was used at room temperature for 1 hr. Immunoblot analysis was performed with horseradish peroxidase- conjugated anti-rabbit or anti-mouse IgG antibodies using enhanced chemiluminescence Western blotting detection reagents (Amersham Biosciences). The quantification of Western blotting results was done by Image Lab statistic software (Bio-red).

### Cell growth assay *in vitro*

To determine cell growth, 2.0×10^4^ LNCaP cells, 1.5×10^4^ CWR22RV1 cells, 6.0×10^3^ C4-2 cells, 5.0×10^3^ PC-3 cells, 5.0×10^3^ DU145 cells were plated in triplicate in 24-well culture plates. Cell medium was replenished and cell growth was determined by MTT assay (Sigma) and direct cell count. Serum-free medium containing MTT (0.5 μg/ml) was added into each well. After two hrs incubation at 37°C all crystals had solubilized and the optical density of the solution was determined spectrophotometrically at 570 nm [33–34].

### Cytotoxicity assay (the IC_50_ value determination) *in vitro*

Cytotoxicity assay was performed according to the protocol in our laboratory [35]. To determine the IC_50_ value, 1.0×10^6^ LNCaP cells, 5.0×10^5^ CWR22RV1 cells, 6.0×10^4^ C4-2 cells, 5.0×10^4^ DU145 cells, and 5.0×10^4^ PC-3 cells were plated in triplicate in 5% CS-FBS RPMI medium in 24-well culture plates. The cells were incubated with serial concentrations of baicalein for 2 days and cell viability was determined in triplicate by MTT assay. Baicalein + medium only (no cells) were included as controls. Baicalein treated cells were compared to untreated cell control wells. IC_50_ values were analyzed with the program CompuSyn (Developer).

### Statistics

Data are presented as the means ± SDs for the indicated number of separate experiments. The statistical significance of differences between two groups of data were analyzed by paired t-test and P-values <0.05 were considered significant.

## CONCLUSION

In conclusion, the results of the present study demonstrated that baicalein, a natural compound isolated from *Scutellaria baicalensis Georgi* extract, is a novel potential AR signaling inhibitor, which can block AR regulated gene expression and cell growth in androgen-sensitive cells and in CRPC cells. The inhibitory effects of baicalein are due to its interference with the AR N-C dimerization and AR-coregulator complex formation. Our data also indicated that baicalein could inhibit the E2 or Adiol-induced AR transactivation in the absence of androgens. These results suggest that baicalein may represent a promising anti-AR compound to better suppress the CRPC.

## Abbreviations

AR: androgen receptor
PCa: prostate cancer
RT-PCR: reverse transcription polymerase chain reaction
FBS: fetal bovine serum
ADT: androgen deprivation therapy
MTT: 3-(4,5-dimethylthiazol-2-yl)-2,5-diphenyltetrazolium bromide
CRPC: castration-resistant prostate cancer
DHT: 5α-dihydrotestosterone
DEX: dexamethasone
HF: hydroxflutamide
E2: 17β-estradiol
DMSO: dimethyl sulphoxide.
